# Reptiles in the One Health ecology of antimicrobial resistance, with insights into zoonotic pathogens: reservoirs, transmission pathways, and public health implications

**DOI:** 10.3389/fmicb.2026.1965652

**Published:** 2026-10-13

**Authors:** Christa Ewers, Franziska Unger, Ellen Prenger-Berninghoff, Tobias Eisenberg

**Affiliations:** 1 Institute of Hygiene and Infectious Diseases of Animals, Justus Liebig University Giessen, Giessen, Germany; 2 Department of Veterinary Medicine, Hessian State Laboratory, Giessen, Germany

**Keywords:** antimicrobial resistance, antimicrobial resistance genes, Enterobacterales, multidrug resistance, One Health, reptiles, *Salmonella*, wildlife

## Abstract

Antimicrobial resistance is a growing global health challenge at the interface of human, animal, and environmental health. Reptiles—including wild populations, pets, farmed species, and internationally traded animals—are increasingly recognized as participants in One Health AMR networks, occupying ecological interfaces that facilitate interactions among wildlife, domestic animals, humans, and the environment. Their diverse microbiota and exposure to both natural and anthropogenically influenced habitats create opportunities for the acquisition and dissemination of antimicrobial-resistant bacteria (AMR bacteria) and antimicrobial resistance genes (ARGs), particularly in captive settings where close human contact and husbandry practices may favor bacterial exchange. Recent studies demonstrate that reptiles frequently harbor clinically relevant ARGs encoding extended-spectrum β-lactamases (ESBL), AmpC-β-lactamases, carbapenemases, and colistin resistance. Potential exposure pathways include direct contact, bites, environmental contamination of terraria and husbandry systems, and, in some regions, the handling and consumption of reptile-derived food products. Wild reptiles may additionally serve as valuable sentinels for anthropogenic AMR pollution within terrestrial and aquatic ecosystems. Despite growing interest, the role of reptiles within the broader ecology of AMR remains incompletely understood. Available studies are geographically uneven, methodologically heterogeneous, and frequently lack high-resolution genomic characterization, limiting robust assessment of transmission pathways, transmission directionality, and the relative contribution of reptiles compared with other wildlife and environmental reservoirs. This review synthesizes current evidence on the occurrence, diversity, and transmission of AMR bacteria in reptiles and discusses their implications for public health, veterinary medicine, wildlife conservation, and One Health. It further highlights major methodological limitations, critical knowledge gaps, and priorities for harmonized, genomics-informed surveillance. Overall, current evidence suggests that reptiles should be viewed as ecologically relevant participants in the One Health ecology of antimicrobial resistance, although their relative contribution to AMR dissemination remains to be quantified.

## 1 Introduction

Antimicrobial resistance (AMR) has emerged as one of the most pressing global health challenges, affecting human and veterinary medicine, agriculture, and environmental health alike. Consequently, AMR is conceptualized within a One Health framework, emphasizing the interconnectedness of humans, animals, and their shared environments. Environmental reservoirs are now considered integral components of AMR ecology because they facilitate the persistence, evolution, and dissemination of resistant bacteria and antimicrobial resistance genes (ARGs) across ecological boundaries (Al-Khalaifah et al., 2025; Bengtsson-Palme et al., 2023; Larsson et al., 2023).

Reptiles represent a particularly interesting but still underexplored component of this network. Whether living in the wild, maintained in captivity, farmed for food production, or traded internationally as pets, reptiles harbor diverse bacterial communities that include numerous opportunistic and zoonotic pathogens, frequently without exhibiting clinical signs of disease (Marin et al., 2020; Valença et al., 2025). Their close contact with humans through pet ownership, zoological collections, breeding facilities, wildlife rehabilitation, and the international animal trade creates multiple opportunities for bacterial exchange (Paphitis et al., 2025). In parallel, exposure to contaminated water, feed, substrates, wildlife, livestock, and anthropogenic waste, and plastic debris may facilitate acquisition and dissemination of resistant bacteria and ARGs within reptile-associated microbiota (Assefa et al., 2025; Eibach et al., 2019; Jourand et al., 2026).

Over the past decade, an increasing number of studies have demonstrated that reptile-associated bacteria may exhibit resistance to clinically important antimicrobials, including multidrug resistance (MDR), extended-spectrum β-lactamase (ESBL)- and AmpC-β-lactamase-mediated resistance to third-generation cephalosporins, and, occasionally, resistance to other last-resort antimicrobials, such as carbapenems and polymyxins (Ahmed et al., 2007; Cortés-Cortés et al., 2016; Goławska et al., 2019; Goldberg et al., 2019; Marin et al., 2020; Unger et al., 2017; Unger et al., 2024; Valença et al., 2025). However, reported prevalence varies considerably among studies due to differences in reptile species, geographic origin, ecological setting, husbandry practices, sampling strategies, bacterial targets, and laboratory methodologies (Muslin et al., 2025; Pees et al., 2023). Consequently, direct comparison of prevalence estimates across studies remains difficult.

From a One Health perspective, reptiles are of particular interest because they occupy ecological interfaces within and between wildlife, domestic animals, humans, and aquatic or terrestrial environments. Several studies have identified ARGs, plasmids, and bacterial lineages in reptiles that overlap with those reported from humans, livestock, and environmental sources, suggesting that reptiles participate in broader ecological networks of AMR dissemination rather than representing isolated reservoirs (Assefa et al., 2025; Choual et al., 2026; Eibach et al., 2019; Jourand et al., 2026). Nevertheless, current evidence is insufficient to infer transmission directionality or to quantify the relative contribution of reptiles compared with other wildlife or environmental reservoirs. Moreover, despite increasing surveillance efforts, global coverage remains uneven, and genomic characterization of reptile-associated AMR remains limited. For example, in two comprehensive reviews of AMR in wildlife, each including more than 200 studies, investigations focusing on reptiles accounted for only a small proportion of the available literature (Li et al., 2024; Torres et al., 2021). These knowledge gaps hamper a robust assessment of the ecological and public health relevance of reptile-associated AMR and limit evidence-based mitigation strategies (Bengtsson-Palme et al., 2023; Delpy et al., 2024).

This review summarizes current knowledge on reptile-associated bacteria, with particular emphasis on antimicrobial resistance, zoonotic relevance, and potential transmission pathways within a One Health framework. Particular attention is given to methodological challenges in AMR surveillance, critical knowledge gaps, and priorities for future research that integrates microbiological, genomic, and epidemiological approaches at the human–animal–environment interface.

## 2 Literature search strategy

To identify relevant studies, a structured literature search was conducted in PubMed from database inception until July 2026 using predefined keywords combined with the Boolean operators “AND” and “OR.” The search was conducted as follows: reptile-related terms (“reptile,” “turtle,” “tortoise,” “terrapin,” “snake,” “lizard,” “gecko,” “iguana,” “crocodile,” “caiman,” “alligator,” “tuatara,” and “amphisbaenian”) linked by “OR” were queried with “AND” in combination with terms regarding antimicrobial resistance (“antimicrobial resistance,” “AMR,” “MDR,” “multidrug resistance,” and “multidrug-resistant”). A search using these terms yielded in 216 results. Inclusion criteria comprised that the samples were from reptiles, bacteria were examined generally at least on phylum level and testing for AMR was conducted. Based on these inclusion criteria, 73 studies could, therefore, be excluded by assessing their titles and abstracts. The database search was supplemented by manual screening of reference lists and targeted searches for relevant recently published or non-PubMed-indexed literature, with the final update performed in July 2026. This additional search identified two further articles. Original research articles, surveillance studies, case reports and review articles were considered eligible. To avoid duplication of data, primary research articles served as the sole basis for quantitative and qualitative synthesis. Review articles and meta-analyses were utilized exclusively for contextualization and to corroborate emerging patterns, ensuring that findings from primary studies were not double-counted. Non-English publications and conference abstracts without full-text availability were excluded. Retrieved records were screened by title, abstract, and full-text assessment according to the described inclusion and exclusion criteria.

Ultimately, 145 publications met the inclusion criteria and were included in this review, comprising 138 primary research articles and seven review articles, two of which were meta-analyses. Primary research articles were classified according to study design, with multiple study types assigned where appropriate. Screening or prevalence studies were by far the most common (*n* = 89), followed by retrospective studies (*n* = 18), case reports (*n* = 16), experimental studies (*n* = 10), clinical case series (*n* = 8), clinical case studies (*n* = 3), outbreak investigations (*n* = 3), and surveillance or monitoring studies (*n* = 3). In addition, one case–control study was identified. Because several publications combined more than one study design, the totals across study categories exceed the number of included primary research articles. Studies investigated captive reptiles (*n* = 57), wild reptiles (*n* = 52), or both captive and wild populations (*n* = 21), whereas the remaining 15 publications comprised review articles, experimental studies, or investigations, in which reptiles represented only one of several animal groups examined, or studies that could not be clearly assigned to either captive or wild populations. *Salmonella* was by far the most frequently investigated bacterial target, being included in 49 publications, of which 40 focused exclusively on *Salmonella* and nine investigated this genus alongside other bacterial taxa. Beyond this taxonomic focus, considerable methodological heterogeneity was evident, with some studies reporting AMR only for broad bacterial groups (e.g., Gram-positive or Gram-negative bacteria) rather than for individual bacterial genera or species. Most studies originated from Europe (*n* = 40) and Asia (*n* = 38), followed by North America (*n* = 23) and South America (*n* = 21), whereas comparatively few investigations were conducted in Africa (*n* = 8) and Oceania (*n* = 3). Eight studies represented global studies that could not be assigned to distinct continents, while another four were multi-country studies that were assigned to at least two different continents.

## 3 Prevalence and diversity of antimicrobial-resistant bacteria in reptiles

The available literature demonstrates substantial heterogeneity regarding host species, sampling strategies, microbiological methods, bacterial targets, and geographic coverage (Supplementary Table S1). Consequently, quantitative comparisons between studies remain difficult, and a formal meta-analysis was considered not appropriate. Rather than providing pooled prevalence estimates, this review synthesizes current evidence on the diversity of antimicrobial-resistant bacteria and acquired resistance genes to identify recurring patterns, major resistance mechanisms, and important knowledge gaps.

Overall, reptiles have consistently been shown to harbor AMR bacteria belonging to diverse taxonomic groups, including Enterobacterales (especially *Salmonella*), *Acinetobacter*, *Aeromonas*, *Enterococcus*, *Pseudomonas*, *Staphylococcus*, and *Vibrio* (Drane et al., 2021; Li et al., 2024; Muslin et al., 2025; Unger et al., 2022). Reported resistance determinants comprise genes mediating resistance to β-lactams, aminoglycosides, fluoroquinolones, tetracyclines, sulfonamides, polymyxins, and other clinically important antimicrobial classes. An overview of the acquired ARGs reported in reptile-associated bacteria is provided in Table 1, whereas detailed study characteristics, bacterial taxa, resistance phenotypes, and study-specific resistance gene profiles are summarized in Supplementary Table S1. However, the available evidence remains strongly influenced by methodological factors. Many investigations specifically targeted *Salmonella* spp. or third-generation cephalosporin-resistant Enterobacterales because of their recognized public health relevance, whereas non-target bacterial taxa were investigated far less frequently. Consequently, current knowledge likely overestimates the prevalence of these priority pathogens while underrepresenting the broader diversity of reptile-associated resistant bacteria.

**Table 1:** Global distribution of acquired antimicrobial resistance genes reported in reptile-associated bacteria.

Resistance class	Studies (*n*)	Reported acquired ARGs	Main bacterial taxa	Main hosts	Setting	Representative regions
β-Lactams: broad spectrum and others	24	*bla*_TEM-1, -1B, -1C_; *bla*_SHV-1_; *bla*_OXA-1, −2, −10, −21-like, −30, −55_; *bla*_P1;_ *bla*_Z_	*E. coli, S. enterica, Klebsiella, Acineto-bacter, Staphylococcus*	Snakes, lizards, turtles, tortoises, sea turtles	Mainly captive; occasional wild and marine	DE, FR; CN, HK, TW; MX, US; BR
β-Lactams: ESBL	25	*bla*_CTX-M-1, −2, −3, −8, −9, −14, −15, −18, −24, −27, −55, −64, −65, −83_; *bla*_SHV-2, −2a, −5, −12, −28, −36_; *bla*_TEM-3, −10, −12, −20, −24, −29, −33, −50, −52, −63, −116, −150, −176, −236_; *bla*_PER-1_	*E. coli, S. enterica, Citrobacter, K. pneu-moniae, Ent. cloacae*	Snakes, lizards, chelonians	Mainly captive; occasional wild and marine	DE, ES, IT, PT, UK; CN, HK, JP, TW; CA, MX, US; BR; GP
β-Lactams: pAmpC	20	*bla*_CMY-1, −2, −4, −12, −13, −23, −26, −37, −42, −61, −83, −98, −117, −129-like, −135_; *bla*_ACT-2, −4, −7, −16, −24_; *bla*_DHA-1, −15_; *bla*_LAT-1_; *bla*_MIR-1_; *bla*_BIL-1_	*E. coli, S. enterica, C. freundii, Ent. cloacae*, other EB	Snakes, turtles, tortoises, sea turtles	Mainly captive; occasional wild and marine	ES, IT; CN, HK, JP; CA, MX, US; BR
β-Lactams: carbapenems	7	*bla*_NDM-1_; *bla*_IMP-27_	*Citrobacter, Provi-dencia, K. pneumoniae, Acinetobacte*r	Turtles, sea turtles, snakes	Captive and wild	IT; CN, IN, KR; BR
Colistin	5	*mcr-1*, *mcr-2, mcr-4*	*E. coli, S. enterica*, other EB	Turtles, snakes, lizards	Captive and wild	ES; CN; CA, US; EC; ZA
Fluoroquinolones	26	*qnrA1*, *qnrB1*,-*2*,*-4*,-*6*,-*9*,-*12*,-*19*; *qnrD; qnrS1*,-*2*,-*13*; *qnrVC4*; *qepA8*, *oqxAB*; *aac(6′)-Ib-cr*; target mutations: *gyrA*, *gyrB*, *parC*, *parE*	EB	Snakes, lizards, chelonians	Captive and wild	DE, IT, UK; CN, HK, JP, TW; CA, MX, US; BR, EC; ZA
Aminoglycosides	35	*aadA1*,-*2*,-*5*,-*16*,-*22*; *aadB*; *aac(3)-IIa*,*-IIe*,*-IId*,*-Iva*; *aac(6′)-Ia*,*-Ib*,*-If*,*-Ir*,*-Iz*; *ant(2″)-Ia*; *aph(2″)-IIIa*, *aph(3′)-Ia*,-*Ib*,*-III*,*-VI*,*-IX*,*-X*; *aph(4)-Ia*, *aph(6)-Id*; *armA*, *rmtB/C*; *strA/B*	EB, *Acinetobacter*, *Stenotrophomonas*	Snakes, lizards, chelonians	Captive and wild	IT; CN, HK, IN, TW; CA, US; BR
Tetracyclines	31	Efflux: *tet(A)*,*-(B)*,*-(C)*,*-(D)*,*-(E)*,*-(G)*,*-(L)*,*-(39)*,*-(45)*; ribosomal protection: *tet(M)*,*-(O)*,*-(Q)*,*-(W)*,*-(32)*,*-(44)*; enzymatic inactivation: *tet(X)*	EB, *Enterococcus*, *Acinetobacter*	Snakes, lizards, chelonians	Captive and wild	DE, ES, IT, LT, PT; CN; CA, MX, US; BR, EC; CX
Sulfonamides	23	*sul1*, *sul2*, *sul3*	EB, *Acinetobacter*	Snakes, turtles, lizards	Captive and wild	DE, IT; CN, TW; CA, MX; BR
Trimethoprim	22	*dfrA1*,-*5*,-*7*,-*10*,-*12*,-*14*,-*17*,-*19*,-*21*,-*27*; *dfrE/K*	EB, *Enterococcus*, *Staphylococcus*	Snakes, turtles, lizards	Captive and wild	SK; CN, TW; CA, MX; BR
Phenicols	19	*catA1*,*-2*; *catB2*,*-3*,*-4*; *cmlA1*,*-5*,*-10*; *floR*; *cfr*	*E. coli, S. enterica, Klebsiella*, *Acinetobacter*	Snakes, chelonians	Mainly captive; occasional wild	CN, HK, TW; CA, MX; BR
Macrolides/Lincosamides	14	*mph(A)*,*-(B)*; *erm(B)*,*-(F)*,*-(42)*,*-(K)*; *lnu(F)*,*-(G)*; *mef(A)*; *msrA/C*	EB, *Enterococcus*, *Staphylococcus*	Snakes, lizards, turtles	Captive	CN, HK, IN; CA, MX; BR
Fosfomycin	9	*fosA*, *fosA7*, *fosX*	EB, *Listeria*	Turtles, tortoises, sea turtles	Captive and wild	IT; CN; US; BR
Rifamycins	4	*arr-2*,*-3*	EB	Turtles, tortoises	Captive	CN; US; BR
QAC/biocides	3	*qacEΔ1*, *qacH*, *qacL*; *bcrRABD*	EB, *P. aeruginosa*, *Enterococcus*	Turtles, snakes, geckos	Mainly captive; occasional wild	FR; CN; GN; CX

Studies (*n*) indicate the number of independent original studies reporting at least one acquired antimicrobial resistance gene within the respective antimicrobial class; individual studies may contribute to more than one class. Only acquired resistance determinants are included; intrinsic chromosomal resistance genes were excluded unless explicitly reported as acquired or plasmid mediated. For space reasons, the designation “spp.” has been omitted throughout the table for taxa identified only to the genus level. Countries are presented using ISO 3166-1 alpha-2 codes and ordered by geographic region to improve readability and conserve space. Detailed study characteristics, references, and study-specific resistance gene profiles are provided in Supplementary Table S1. *C*.: *Citrobacter*; *E*.: *Escherichia*; EB: Enterobacterales; *Ent*.: *Enterobacter*; *K*.: *Klebsiella*; *P*.: *Pseudomonas*; *S*.: *Salmonella*.

In addition, surveillance efforts remain geographically uneven, with most studies originating from Europe and Asia, a moderate body of evidence from North and South America, and comparatively little information available from Africa and Oceania. Genomic characterization has also become increasingly common only during recent years, meaning that older studies frequently report phenotypic resistance without molecular characterization of the underlying resistance determinants. Accordingly, the available literature should primarily be interpreted as evidence for the diversity and occurrence of AMR bacteria in reptiles rather than as a basis for estimating their quantitative contribution to the global burden of AMR.

### 3.1 Captive reptiles

Captive reptiles—including pet animals, zoological collection animals, ex situ breeding stock in conservation facilities, and farmed reptiles—are exposed to ecological conditions that differ fundamentally from those experienced by free-ranging populations. High animal densities, shared enclosures, repeated transport, frequent contact with humans and other animal species, and the prolonged persistence of bacteria in water systems and biofilms facilitate bacterial transmission and long-term colonization (Cristina et al., 2022; Marin et al., 2020; Muñoz-Ibarra et al., 2022). In addition, reptiles maintained under human care or in human surroundings may be exposed to antimicrobial residues through contaminated environments or receive antimicrobial treatment during veterinary care, thereby creating selective pressures that favor the emergence, persistence, and dissemination of AMR bacteria (Barbosa et al., 2023).

Although antimicrobial therapy is increasingly applied in reptile medicine, evidence-based treatment protocols remain limited (Chuliuk, 2021; Eatwell, 2007). In clinical practice, antimicrobial selection, dosage regimens, and treatment duration are frequently extrapolated from humans or other domestic animal species, because reptile-specific pharmacokinetic and pharmacodynamic data are scarce (Barbosa et al., 2023; Jelinski et al., 2022; Loeb, 2024). Such extrapolation may contribute to suboptimal antimicrobial exposure and the selection of resistant bacteria, emphasizing the importance of culture-based diagnostics and antimicrobial susceptibility testing whenever feasible before initiating therapy (Brockmann et al., 2020; Cristina et al., 2022; Hedley et al., 2021). The impact of antimicrobial exposure is illustrated by comparative studies in green sea turtles, in which captive animals more frequently harbored bacteria resistant to antimicrobials commonly used during rehabilitation, whereas resistance profiles in free-ranging turtles more closely reflected environmental exposure (Ghafoor et al., 2025b).

Among reptile-associated bacteria, members of the Enterobacterales have received by far the greatest attention because of their importance as zoonotic pathogens and their frequent association with clinically relevant antimicrobial resistance. Studies from Europe, Asia, Africa, North and South America demonstrate that captive reptiles frequently harbor multidrug-resistant (MDR), extended-spectrum β-lactamase (ESBL)-producing, and AmpC-producing Enterobacterales, particularly *Salmonella enterica*, *Escherichia coli*, *Klebsiella pneumoniae*, *Citrobacter* spp., *Enterobacter* spp., *Providencia* spp., and *Proteus* spp. (Ahmed et al., 2007; Bertelloni et al., 2016; Chen et al., 2010; Cortés-Cortés et al., 2016; Da Silva et al., 2025; Goławska et al., 2019; Guyomard-Rabenirina et al., 2020; Hossain et al., 2020; Marin et al., 2020; Morrison and Rubin, 2020; Song et al., 2023; Valença et al., 2025; Waltenburg et al., 2022; Wang et al., 2024).

Historically, the first ESBL identified in reptile-derived Enterobacterales was *bla*_SHV-36_, detected in a captive tortoise in Japan (Ahmed et al., 2007). Since then, a broad spectrum of clinically important β-lactamase genes has been reported, including members of the *bla*_CTX-M_, *bla*_SHV_, *bla*_TEM_, *bla*_CMY_, and *bla*_DHA_ families, together with rarely detected carbapenemase genes such as *bla*_IMP-27_ (Colon et al., 2022; Cortés-Cortés et al., 2016; Da Silva et al., 2025; Deng et al., 2016; Goławska et al., 2019; Guyomard-Rabenirina et al., 2020; Hossain et al., 2020; Li et al., 2024; Mengistu et al., 2022; Morrison and Rubin, 2020; Pathirana et al., 2018; Ríos et al., 2022; Trotta et al., 2021; Unger et al., 2024; Valença et al., 2025; Waltenburg et al., 2022; Wang et al., 2024). In addition, several investigations identified chromosomally encoded AmpC β-lactamases and OXA-type enzymes, illustrating the diversity of β-lactam resistance mechanisms associated with captive reptiles (Dias et al., 2014; Liu et al., 2017; Trotta et al., 2021).

Multidrug-resistant *Salmonella* has repeatedly been recovered from captive reptiles, although reported MDR prevalence varied markedly among studies, ranging from approximately 6% to more than 70% depending on reptile species, geographic origin, husbandry conditions, sampling strategy, and laboratory methodology (Bertelloni et al., 2016; Chen et al., 2010; Marin et al., 2020; Song et al., 2023). In Lithuania, *Salmonella* was detected in approximately 61% of sampled captive reptiles, and 20% of the recovered isolates were MDR; ARGs mediating resistance to tetracyclines, aminoglycosides, sulfonamides, and trimethoprim were also identified (Merkevičienė et al., 2022). Likewise, third-generation cephalosporin-resistant Enterobacterales carrying *bla*_CTX-M-15_ and *bla*_CMY-2_ have been detected in captive leopard geckos and bearded dragons (Valença et al., 2025), further demonstrating that clinically relevant β-lactam resistance mechanisms are not restricted to *Salmonella,* but occur across different Enterobacterales species. Overall, the considerable heterogeneity reported between studies most likely reflects methodological differences and variation in reptile populations rather than genuine biological differences in the occurrence of antimicrobial resistance.

Beyond β-lactam resistance, reptile-associated Enterobacterales frequently harbor genes conferring resistance to tetracyclines (*tet*), sulfonamides (*sul*), aminoglycosides (*aad, aac, aph, ant, armA, rmtB/C, strA/B*), trimethoprim (*dfrA*), plasmid-mediated quinolone resistance determinants (*qnr, qepA, oqxAB, aac(6′)-Ib-cr*), phenicols (*cat, cml, floR*), and macrolides/lincosamides [*erm, mph, Inu, mef, msr* (Table 1; Supplementary Table S1)]. Several studies have further identified class 1 integrons and conjugative plasmids, and other mobile genetic elements, highlighting the potential for horizontal gene transfer (HGT) within reptile-associated bacterial communities. Nevertheless, the diversity and prevalence of these mobile resistance determinants are likely underestimated, because many earlier investigations relied on targeted PCR assays rather than whole-genome sequencing or other comprehensive genomic approaches.

Besides Enterobacterales, numerous opportunistic bacterial pathogens exhibiting clinically relevant resistance phenotypes have been recovered from captive reptiles. Frequently reported genera include *Pseudomonas*, *Acinetobacter*, *Staphylococcus*, *Enterococcus*, *Stenotrophomonas*, *Morganella*, *Citrobacter*, and *Proteus*, several of which display multidrug-resistant phenotypes (Chen et al., 2026; Choual et al., 2026; Cristina et al., 2022; Sala et al., 2019; Strompfová et al., 2024; Unger et al., 2022). Particularly noteworthy is the high prevalence of MDR *P. aeruginosa* in captive snakes, methicillin-resistant biofilm-forming staphylococci colonizing reptile skin (Strompfová et al., 2024), and *Acinetobacter* isolates carrying novel OXA β-lactamases (Unger et al., 2022). Together, these findings demonstrate that AMR in captive reptiles extends far beyond the traditionally investigated Enterobacterales.

Whole genome sequencing studies have considerably expanded current knowledge of reptile-associated AMR. Imported reptiles were shown to carry ESBL- and AmpC-producing Enterobacterales harboring clinically important ARGs including *bla*_CTX-M-15_, *bla*_CTX-M-27_, *bla*_CMY-1/2_, and occasionally plasmid-mediated colistin resistance genes of the *mcr* family (including *mcr-1*, *mcr-2*, and *mcr-4*) as well as internationally disseminated high-risk clones such as *E. coli* ST131 and *K. pneumoniae* ST307 (Foster et al., 2020; Schmidt et al., 2024; Unger et al., 2024). Likewise, ESBL-producing *E. coli* belonging to ST155 and ST224 carrying *bla*_CTX-M-8_ together with *tet*(A), *sul2*, and *dfrA* were recovered from captive Amazonian mud turtles (Da Silva et al., 2025). Additional reports describing MDR *Providencia rettgeri* carrying *bla*_IMP-27_ and β-lactamase-producing *Aeromonas* spp. further illustrate that reptiles may harbor bacterial lineages and resistance mechanisms of considerable clinical relevance (Carvalho et al., 2018; Pathirana et al., 2018).

Collectively, current evidence indicates that captive reptiles frequently harbor multidrug-resistant bacterial lineages encompassing both commensal and opportunistic pathogens. However, current knowledge is strongly influenced by methodological bias, as most studies specifically targeted *Salmonella* spp. or Enterobacterales, because of their clinical and regulatory relevance. Consequently, these bacterial groups and their associated resistance mechanisms are likely overrepresented, whereas resistance in non-target taxa remains considerably underexplored. In addition, the available data do not permit conclusions regarding the direction of transmission, nor do they indicate that reptiles may serve as contributors to the broader AMR landscape of these resistance determinants. Instead, the observed overlap most likely reflects the shared ecological networks, in which humans, domestic animals, wildlife, and environmental bacterial communities continuously exchange microorganisms and ARGs. Future investigations should combine standardized culture-based methods with whole-genome sequencing and metagenomic approaches to obtain a more comprehensive understanding of reptile-associated microbiomes and resistomes as well as their role within the broader ecology of AMR. In addition to the bacterial taxa highlighted above, AMR has also been reported in numerous other genera, including *Vibrio*, *Bordetella*, *Chlamydia*, and several other taxa (Chaiwattanarungruengpaisan et al., 2024; Choual et al., 2026; Esposito et al., 2024; Jourand et al., 2026; Li et al., 2018; Pathirana et al., 2018; Ramos et al., 2019; Tsai et al., 2021; Tsai et al., 2024; Zając et al., 2025; Zavala-Norzagaray et al., 2015).

### 3.2 Wild reptiles

Unlike captive reptiles, free-ranging reptiles are rarely exposed to direct antimicrobial treatment. Although naturally occurring resistance mechanisms are also present in environmental bacteria, the occurrence of clinically relevant antimicrobial resistance genes is widely regarded as an indicator of anthropogenic influence on natural ecosystems. Depending on their ecology, wild reptiles may acquire AMR bacteria through contact with soil, freshwater and marine environments, sediments, prey species, livestock, wastewater, or other anthropogenically influenced habitats. As a result, wild reptiles represent valuable sentinel species for assessing the dissemination of AMR at the wildlife–environment interface within a One Health framework (Arnold et al., 2016; Di Nicola et al., 2025; Guyomard-Rabenirina et al., 2020; Nieto-Claudin et al., 2021; Tsai et al., 2024).

As in captive reptiles, members of the Enterobacterales, particularly *Salmonella enterica* and *E. coli*, represent the best investigated bacterial groups in wild reptile populations. Phenotypic resistance has been reported in snakes, lizards, tortoises, turtles, and crocodilians from several continents, although reported AMR prevalence varies considerably between studies, ranging from no detectable resistance to more than 70% of isolates (Abrahão et al., 2020; Doden et al., 2021; La Tela et al., 2021; Małaszczuk et al., 2025; Marin et al., 2022; McWhorter et al., 2021; Molina-López et al., 2015; Santana-Hernández et al., 2023; Sumiyama et al., 2020a; Sumiyama et al., 2020b). Several additional investigations reported phenotypic resistance profiles without further genomic characterization, with resistance rates ranging from <4 to 100%, further illustrating the considerable methodological heterogeneity of the available literature (Padhi et al., 2020; Ramos et al., 2019; Rasmussen et al., 2017).

Molecular investigations further demonstrate that wild reptiles may harbor clinically relevant resistance determinants. *Salmonella* isolates from free-ranging snakes, lizards, and tortoises carried plasmid-mediated quinolone resistance determinants including members of the *qnr* family (*qnrA*, *qnrB*, and *qnrS*), together with aminoglycoside resistance genes such as *strA* (Mlangeni et al., 2024; Mlangeni et al., 2025). Likewise, ESBL- and AmpC-producing Enterobacterales carrying *bla*_CTX-M-15_, *bla*_CMY-1_, and *bla*_CMY-2_ have been detected in free-ranging red-eared sliders and sympatric mammalian wildlife in Spain, suggesting exposure to shared environmental hosts harboring clinically relevant ARGs rather than host-specific circulation (Mengistu et al., 2022). Similarly, wild iguanas (*Iguana* spp.) and anoles (*Anolis* spp.) from Guadeloupe carried ESBL-producing *E. coli* harboring *bla*_CTX-M-1_, *bla*_TEM-52_, and *bla*_TEM-50_, together with IncF plasmids frequently associated with the dissemination of clinically relevant ARGs (Guyomard-Rabenirina et al., 2020; Smoglica et al., 2026).

Beyond Enterobacterales, AMR bacteria have been described in a broad range of bacterial taxa associated with wild reptiles. These include *Aeromonas* spp. harboring diverse β-lactamase genes belonging to the OXA and FOX families (Dias et al., 2014), “*Enterococcus lacertideformus”* carrying *tet(M)*, *sat4*, *dfrE*, and the *bcrRABD* operon (Agius et al., 2021), and *Stenotrophomonas maltophilia* carrying aminoglycoside resistance genes (Li et al., 2023). Although molecular investigations remain comparatively scarce, these findings indicate that AMR in wild reptiles is not restricted to Enterobacterales but encompasses diverse environmental and opportunistic bacterial species.

Marine reptiles provide particularly valuable insights into the dissemination of AMR within coastal ecosystems because they integrate microbial exposure over large geographic areas and extended time periods. Green sea turtles (*Chelonia mydas*) admitted to rehabilitation centres have yielded ESBL-producing *Ent*. *hormaechei* and *C. freundii* carrying *bla*_CTX-M-15_, together with *fosA*, *qnrB1*, *sul*, *tet*(A), and heavy-metal resistance determinants, reflecting the influence of anthropogenic contamination on coastal microbiomes (Ghafoor et al., 2025a; Ghafoor et al., 2025b; Goldberg et al., 2019). Additional multidrug-resistant Enterobacterales, including *Klebsiella* spp. and *Salmonella* Infantis, have recently been recovered from sea turtles at admission to rehabilitation centres, further supporting the value of rehabilitation facilities as surveillance sites for monitoring AMR in free-ranging marine reptiles (Smoglica et al., 2026). Particularly noteworthy is the recent emergence of carbapenemase-producing Enterobacterales in marine turtles. In Brazil, a diseased green sea turtle yielded *C. freundii* harboring *bla*_CMY-117_ and *bla*_NDM-1_ (Aravena-Ramírez et al., 2024), while a multidrug-resistant *C. portucalensis* isolated from an olive ridley sea turtle (*Lepidochelys olivacea*) carried *bla*_NDM-1_, *bla*_CMY-13_, and numerous additional ARGs (Sellera et al., 2023). More recently, an NDM-1-producing *Citrobacter* spp. isolate carrying additional resistance genes was reported from a loggerhead sea turtle (*Caretta caretta*), further demonstrating that clinically relevant carbapenemases are no longer exceptional findings in marine reptiles (Fratini et al., 2026). Additional studies identified Enterobacterales, *Pseudomonas* spp., and *Aeromonas hydrophila* carrying β-lactamase genes including *bla*_CTX-M-3_, *bla*_CMY-37_, *bla*_SHV-12_, *bla*_TEM-236_, and chromosomal *bla*_ACT_ variants in loggerhead and olive ridley sea turtles (Foti et al., 2009; Jorge et al., 2023; Trotta et al., 2021; Tsai et al., 2024). Further investigations have reported phenotypically resistant bacteria from sea turtles without molecular characterization, indicating that the actual diversity of resistance determinants in marine reptiles is likely underestimated (Ahasan et al., 2017; Al-Bahry et al., 2011; Tsai et al., 2021).

Compared with other marine reptiles, such as sea snakes and marine iguanas, sea turtles have been investigated considerably more frequently. This discrepancy most likely reflects their protected conservation status, frequent admission to wildlife rehabilitation centres due to boat injuries, and routine microbiological examinations performed during clinical care rather than biological differences in AMR occurrence. Consequently, current knowledge of AMR in marine reptiles is strongly biased toward sea turtles, whereas data for other marine reptile taxa remain extremely limited (Hamann et al., 2010).

Collectively, the available evidence indicates that wild reptiles primarily reflect environmental acquisition of antimicrobial-resistant bacteria and ARGs rather than direct antimicrobial selection pressure. At the same time, the detection of ESBL-, AmpC-, and even carbapenemase-producing bacteria in free-ranging reptiles demonstrates that clinically important AMR has become established beyond human and livestock settings. Future studies combining standardized culture-based methods with whole-genome sequencing and metagenomics will be essential to better define the ecological role of wild reptiles within the environmental resistome and the broader One Health transmission network. Consequently, wild reptiles should be regarded not only for their potential to harbor AMR bacteria but also as valuable bioindicators for monitoring the environmental dissemination of clinically relevant resistance genes across terrestrial and aquatic ecosystems. Marine plastic debris may further contribute to this process by providing surfaces that support the persistence and dissemination of antimicrobial-resistant bacteria. Plastic surfaces are rapidly colonized by microbial communities that form biofilms, collectively referred to as the “plastiphere,” which can harbor potentially pathogenic bacteria and AMR genes. The high cell density and close spatial proximity of bacteria within these biofilms may facilitate horizontal gene transfer, while floating plastics can transport associated microorganisms across marine environments (Ormsby and Quilliam, 2026; Sathicq et al., 2021; Stevenson et al., 2024). This pathway may be particularly relevant for sea turtles, which are frequently exposed to and may ingest plastic debris. Recent evidence of dense communities of potentially pathogenic and antimicrobial-resistant bacteria on marine plastics, together with the detection of closely related strains in plastics and sea turtles, further supports a potential role of plastic debris in the environmental dissemination of AMR (Jourand et al., 2026).

### 3.3 Methodological challenges in reptile AMR surveillance

Assessment of antimicrobial resistance in reptiles is associated with substantial methodological and interpretive challenges that limit comparisons among studies and complicate the interpretations of published findings. One major source of heterogeneity is the wide variety of microbiological methods employed, including the use of selective and non-selective culture media (Supplementary Table S1), which markedly influence the bacterial taxa and resistance determinants detected. For example, studies investigating ESBL- and AmpC-producing Enterobacterales in captive and wild reptiles frequently apply selective screening for third-generation cephalosporin resistance. However, reported prevalence rates, bacterial diversity, and ARG profiles vary considerably depending on the culture media, selective agents, and laboratory protocols used, thereby limiting direct comparisons across studies and geographic regions (Cortés-Cortés et al., 2016; Da Silva et al., 2025; Valença et al., 2025).

A second major challenge is the absence of reptile-specific interpretive criteria for antimicrobial susceptibility testing (AST). For most host-pathogen-antimicrobial combinations, neither validated clinical breakpoints nor species-specific susceptibility thresholds are currently available. Thus, AST results are generally interpreted using clinical breakpoints established for homeothermic animals or, where available, epidemiological cut-off values (ECOFFs), which distinguish wild-type populations lacking acquired resistance mechanisms from non-wild-type isolates with reduced susceptibility. Although these surrogate criteria facilitate standardized reporting, they do not account for reptile-specific pharmacokinetics, pharmacodynamics, or clinical outcomes and may therefore lead to misclassification of resistance phenotypes. Similar limitations have been recognized more broadly in veterinary AMR surveillance, where the lack of host-, species- and tissue-specific breakpoints frequently necessitates extrapolation from unrelated animal species or from humans.

At present, both the Clinical and Laboratory Standards Institute (2024) and the European Committee on Antimicrobial Susceptibility Testing (2025) provide veterinary interpretative criteria for selected bacterial species and hosts, but neither organization currently offers breakpoints specifically developed for reptiles. Given the extraordinary taxonomic diversity of reptiles, comprising more than 12.500 recognized species (Uetz et al., 2026), the establishment of species-specific breakpoints for all taxa is unlikely to be achievable in the foreseeable future. A more realistic approach would be the consistent reporting of quantitative susceptibility data, particularly minimum inhibitory concentrations (MICs), together with the interpretive criteria applied. Such reporting would substantially improve transparency, facilitate future re-interpretation as new breakpoints become available, and enhance comparability among studies employing different interpretive standards. Additional methodological heterogeneity arises from differences in study design, sampling strategies, bacterial target species, and molecular approaches. As discussed in the preceding sections, many studies have focused on selected bacterial taxa or the targeted detection of specific antimicrobial resistance genes, limiting a comprehensive characterization of the reptile-associated resistome. In studies investigating the prevalence of AMR in reptiles, agar disc diffusion has remained a commonly used method for antimicrobial susceptibility testing, although it is generally considered less precise and provides less quantitative information than minimum inhibitory concentration (MIC) determination. MIC-based approaches, however, have been increasingly employed in recent years (see also Supplementary Table S1). At the same time, genomic approaches, including whole-genome sequencing analyses, have gained increasing importance. Their broader application, together with shotgun metagenomics could help overcome some of the limitations of culture- and target-based approaches by enabling a more comprehensive, and in some cases culture-independent, assessment of microbial communities, antimicrobial resistance genes, and mobile genetic elements.

Likewise, MDR has been defined inconsistently across the studies reviewed, making comparisons difficult unless a standardized framework is applied. One such framework was proposed by Magiorakos et al. (2012), which is documented in column U of Supplementary Table S1. According to this definition, for example, Enterobacterales are classified as MDR if they are non-susceptible to at least one antimicrobial agent in three or more antimicrobial classes.

Collectively, these methodological limitations highlight the need for greater harmonization of sampling strategies, culture conditions, antimicrobial susceptibility testing, molecular methods, and reporting standards in reptile AMR surveillance. Interpretation of the available data should therefore be undertaken with appropriate caution, particularly when drawing conclusions regarding prevalence estimates or public health significance. Furthermore, the current lack of standardized, methodologically comparable multi-host datasets precludes robust quantitative comparisons between reptiles and other wildlife, livestock, companion animals, or environmental reservoirs. Consequently, this review does not attempt to rank the relative One Health importance of reptiles among potential AMR reservoirs but instead provides a comprehensive synthesis of the currently available evidence as a foundation for future comparative investigations.

## 4 Transmission of antimicrobial-resistant bacteria between reptiles and humans

The detection of human-associated bacterial lineages, ARGs and plasmid backgrounds in reptiles, particularly in anthropogenically influenced environments, suggests that transmission of AMR bacteria between reptiles and humans may occur. However, with the exception of *Salmonella*, current evidence remains insufficient to determine the predominant directionality of transmission. As this review does not include formal source-attribution analyses, reptiles may function either as recipients or as disseminators of antimicrobial-resistant bacteria depending on the ecological and epidemiological context. Resolving transmission dynamics will require paired, longitudinal, and high-resolution genomic studies. Selected studies documenting confirmed or strongly supported reptile-associated bacterial infections in humans are summarized in Table 2. Figure 1 illustrates the currently hypothesized transmission pathways and should be interpreted as a conceptual framework rather than a weighted network, as the relative contributions and directionality of individual pathways remain unknown.

**Table 2:** Selected studies documenting confirmed or strongly supported reptile-associated bacterial infections in humans.

Reptile exposure	Pathogen(s)	Human infection/evidence of reptile association	Country	Reference
Unknown snakebite	*Escherichia coli*	Snakebite-associated wound infection; *bla*_NDM-11_ positive MDR *E. coli* isolate	India	Rahman et al., 2018
Chinese cobra bite	*Shew. algae*, *M. morganii*	Polymicrobial soft-tissue infection after a snakebite; *bla*_OXA-55_ and *qnrA* in *Shew. algae*, *bla*_DHA-1_ in *M. morganii*	Taiwan	Huang et al., 2021
Chinese cobra bite	Mainly *Enterococcus faecalis, M. morganii*	53/195 (27.2%) patients developed snakebite-associated wound infections	Taiwan	Yeh et al., 2021
Crocodile bite	Mainly *P. aeruginosa*, *Citrobacter* spp., CNS	8/11 (72.7%) snakebite wounds were culture-positive, yielding 21 aerobic bacterial isolates; 18/21 (85.7%) isolates were MDR	Ethiopia	Alelign et al., 2022
Multiple snakebites (mostly *Bothrops* spp.)	*Aer. hydrophila*	155/326 (47.5%) patients developed secondary infections after snakebites; *A. hydrophila* was cultured from 4/7 patients	Brazil	Soares et al., 2023
Savannah monitor lizard bite	*P. aeruginosa*	Septic arthritis; *P. aeruginosa* was recovered from the infected joint	USA	Tehrani et al., 2008
Direct or environmental contact to turtles, lizards and snakes	*Salmonella enterica*	25 multistate outbreaks within RAAS dataset accounted for 1,227 human illnesses; linkage based on culture-confirmed infection plus PFGE and epidemiologic, laboratory and/or traceback evidence	USA	Waltenburg et al., 2022
Direct or environmental contact to crested and leopard geckos, turtles and snakes	*Salmonella enterica*	Six multistate outbreaks comprised 337 human cases; outbreak attribution was epidemiologic/molecular; WGS primarily used to characterize human isolates	USA	Frey et al., 2024
Direct or environmental contact to pet turtles	*Salmonella* Thompson	Genomic comparison of 19 turtle and 28 child isolates from same region and year identified phylogenetically close turtle-human clusters (<20 SNPs), supporting evidence for interspecies transmission; 80.4% of turtle isolates were MDR	China	Wang et al., 2024
Consumption of dried rattlesnake meat	*Salmonella* spp.	*Salmonella* bacteremia with aortic valve endocarditis in a patient regularly consuming dried rattlesnake meat; reptile origin strongly suspected from exposure history, not microbiologically confirmed	USA	Mishra et al., 2021
Direct or environmental contact to lizards, snakes and turtles/tortoises	*Salmonella enterica*	13 human cases WGS-linked to a *S.* I 4,[5],12:i:- outbreak associated with snakes/feeder rodents and 2 to an *S.* Vitkin outbreak associated with bearded dragons	Canada	Paphitis et al., 2025

*Aer*., *Aeromonas*; AMR, antimicrobial resistance; CNS, coagulase negative staphylococci; *M*., *Morganella*; MDR, multidrug resistant; *P*., *Pseudomonas*; PFGE, pulsed-field gel electrophoresis; RAAS, reptile- and amphibian-associated salmonellosis; *Shew*., *Shewanella*; SNP, single-nucleotide polymorphism; WGS, whole-genome sequencing. AMR information is included only where it is central to the documented human infection.

**Figure 1:**
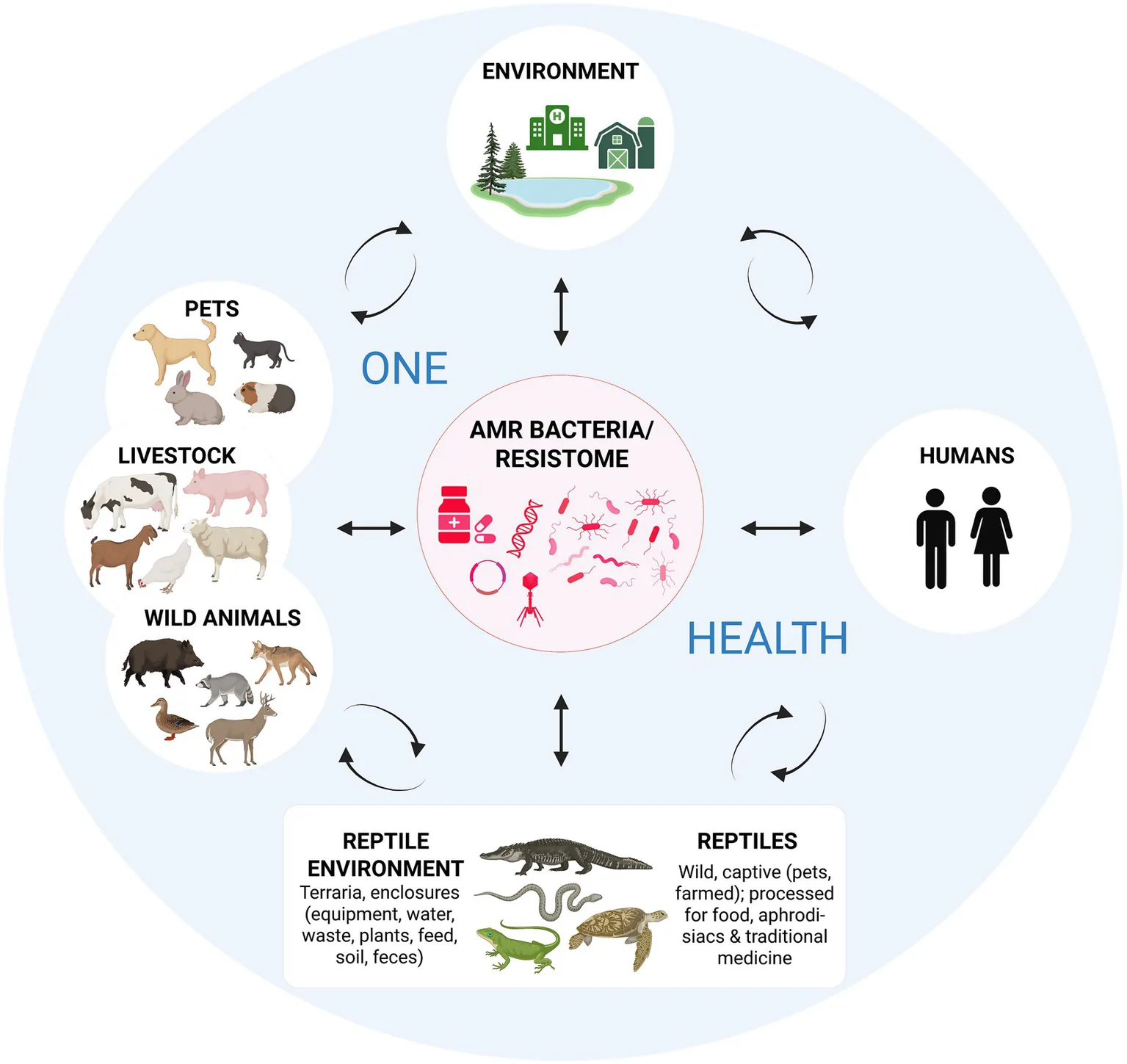
Proposed One Health framework illustrating potential transmission pathways of antimicrobial-resistant bacteria and ARGs involving reptiles. Bidirectional arrows denote hypothesized connectivity and do not imply transmission direction, transmission frequency or relative reservoir importance. Created with Biorender.com.

### 4.1 Direct transmission

Reptiles are well-recognized sources of zoonotic bacterial infections through direct contact, including handling, bites, scratches, or contact with contaminated oral secretions and feces. While *Salmonella* remains by far the best-characterized reptile-associated zoonotic pathogen, increasing evidence indicates that a broader range of bacteria, including antimicrobial-resistant organisms, may also be transmitted through direct reptile contact (Ebani, 2017).

For *Salmonella*, population-based surveillance provides particularly strong epidemiological evidence for the relevance of reptile exposure to human infection. In Ontario, Canada, 6.3% (513/8,148) of sporadic, locally acquired salmonellosis cases with available exposure information and no reported recent travel reported reptile or amphibian contact in the week before symptom onset during 2015–2022 (Paphitis et al., 2025). Reptiles accounted for the predominant reported exposures, particularly lizards, snakes, and turtles or tortoises, and 29.2% of exposed case-patients were <10 years of age. Specific *Salmonella* serotypes were associated with particular reptile groups, including *S. paratyphi* B biovar Java with snakes, *S*. Agbeni with turtles, and *S*. Cotham, *S*. Chester, and *S*. Tennessee with bearded dragons. Although such epidemiological associations do not establish reptiles as the causative source in individual sporadic cases, they further substantiate the public relevance of reptile-associated salmonellosis (Paphitis et al., 2025).

Outbreak surveillance provides complementary evidence. Multistate reptile- and amphibian-associated *Salmonella* outbreaks have repeatedly been documented in the United States, with children <5 years of age being overrepresented among cases (Waltenburg et al., 2022). More recent analysis of animal-contact-associated *Salmonella* outbreaks during 2015–2018 identified six outbreaks linked to reptiles; WGS substantially increased the amount of information available on AMR among the associated human outbreak isolates (Frey et al., 2024). Certain reptile-associated serotypes may also be of particular clinical relevance. *Salmonella* Panama has a well-described ecological association with reptiles, which represent an important non-human source of infection, particularly in parts of Latin America, and has been associated with invasive human infections including septicemia, meningitis, and osteomyelitis (Pulford et al., 2019). In a nationwide Dutch surveillance study, *S. Panama* and *S*. Poona were among the serotypes displaying the highest invasiveness relative to their overall occurrence in human salmonellosis cases (Mughini-Gras et al., 2020).

Among these, *P. aeruginosa* has repeatedly been implicated in wound and joint infections following reptile bites, including septic arthritis after a Savannah monitor-lizard (*Varanus exanthematicus*) bite (Tehrani et al., 2008). Other *Pseudomonas* spp. are common members of the oral and cloacal microbiota of snakes and lizards, and MDR *P. aeruginosa* have been isolated from captive reptiles in several surveillance studies (Dégi et al., 2023; Sala et al., 2019). Importantly, molecular typing demonstrated closely related *P. aeruginosa* isolates in captive snakes and their keepers, providing evidence that bacterial exchange between reptiles and humans can occur during routine animal handling (Colinon et al., 2010).

Clinical studies investigating secondary bacterial infections following snakebite consistently identify members of the reptile oral microbiota as important opportunistic pathogens. *Aeromonas* spp., particularly *A. hydrophila*, are among the most frequently isolated bacteria from snakebite-associated wound infections, and several isolates exhibited reduced susceptibility to commonly used first-line antimicrobials (Jorge et al., 1998; Soares Coriolano Coutinho et al., 2023). Comparable observations have also been reported from Ethiopia, where Alelign et al. recovered a diverse spectrum of Gram-positive and Gram-negative bacteria from infected crocodile bite wounds, with 85.7% of isolates classified as MDR and ciprofloxacin resistance detected in 83% of Gram-negative isolates (Alelign et al., 2022). Likewise, *Shewanella* spp., naturally associated with aquatic environments, have repeatedly been recovered from wound infections complicating cobra bites, demonstrating that environmental bacteria colonizing the reptile oral cavity may cause clinically relevant human infections (Liu et al., 2012). In a cohort of 195 patients with *Naja atra* bites in Taiwan, 27.2% developed wound infections, with *Enterococcus faecalis* and *Morganella morganii* representing the predominant cultured bacteria (Yeh et al., 2021). A genomically characterized *Naja atra* bite-wound infection further demonstrated coinfection with *Shewanalle algae* and *M. morganii*; WGS identified *bla*_OXA-55_ and *qnrA* in *Shewanella algae* and class C β-lactamase genes *bla*_DHA-1_ and *ampH* in *M. morganii* (Huang et al., 2021; Yeh et al., 2023). Several studies further identified *M. morganii*, as well as *Proteus* spp. and other Enterobacterales as common pathogens associated with snakebite wounds, with some isolates displaying resistance to empirically administered antimicrobial agents (Soares Coriolano Coutinho et al., 2023; Yeh et al., 2021, 2023). In addition, *Staphylococcus* spp. from reptile skin or mucosa may carry methicillin resistance determinants or β-lactamase genes, representing a potential zoonotic hazard (Ebani, 2024). Collectively, these case reports and clinical studies provide convincing temporal and epidemiological evidence linking reptile exposure with subsequent human infection, although the causative bacteria have rarely been characterized using high-resolution genomic approaches (Jorge et al., 1998; Liu et al., 2012; Soares Coriolano Coutinho et al., 2023; Tehrani et al., 2008).

A particularly noteworthy case was reported from India, where a human snakebite wound infection caused by an *E. coli* isolate carrying the novel carbapenemase variant NDM-11 was described (Rahman et al., 2018). Although such reports remain rare, they illustrate the potential for reptile-associated wound infections to involve bacteria harboring clinically highly relevant resistance determinants. Despite these observations, high-resolution molecular confirmation of direct transmission remains limited for most non-*Salmonella* pathogens. For *Salmonella*, evidence for zoonotic transmission is considerably stronger and derives from population-based surveillance and outbreak investigations (Paphitis et al., 2025; Waltenburg et al., 2022), complemented by molecular subtyping and increasingly WGS-based analyses (Frey et al., 2024; Wang et al., 2024). In contrast, evidence for other bacterial taxa is still dominated by clinical case reports, epidemiological associations, and culture-based characterization, with only few studies incorporating genomic analyses and even fewer directly comparing reptile and human isolates (Alelign et al., 2022; Soares Coriolano Coutinho et al., 2023; Tehrani et al., 2008; Yeh et al., 2023). Nevertheless, comparative genomic analyses of *Campylobacter fetus* provide an important exception. Studies including both reptile- and human-derived *C. fetus* ssp. *testudinum* isolates have demonstrated the distinct genomic characteristics of this reptile-associated lineage and support its zoonotic potential (Gilbert et al., 2016; Paintsil et al., 2025).

Taken together, current evidence indicates that direct transmission of AMR bacteria from reptiles to humans is biologically plausible and, for several bacterial species, supported by clinical case reports and epidemiological investigations. Furthermore, reptile-associated isolates frequently harbor antimicrobial resistance genes and plasmid backgrounds that are also widespread among human clinical isolates, suggesting the potential for HGT within shared ecological niches (Eibach et al., 2019). However, for most bacterial taxa, the frequency, directionality, and public health relevance of transmission remain poorly quantified. This uncertainty is also reflected in broader epidemiological evidence on AMR acquisition: in a systematic meta-analysis, lizard and frog ownership was not significantly associated with colonization by third-generation cephalosporin-resistant Enterobacterales compared with non-pet ownership (risk ratio 2.34, 95% CI 0.33–16.63), although the wide confidence interval highlights the very limited underlying evidence base (Hackmann et al., 2021).

Addressing these knowledge gaps will require integrated One Health studies combining longitudinal sampling of reptiles and exposed humans with whole-genome sequencing and detailed epidemiological data. In addition, improved understanding of the reptile skin and gastrointestinal microbiome—which strongly influences colonization, persistence, and exchange of AMR bacteria—will further clarify the ecological mechanisms underlying reptile-associated transmission (Khan et al., 2021).

### 4.2 Indirect and environmental transmission

In addition to direct contact, reptiles can contribute to human exposure to AMR bacteria through indirect and environmental pathways. Transmission may occur via contaminated terrarium substrates, water bowls, feeding equipment, enclosure surfaces, and other fomites exposed to feces or oral secretions. Observational studies demonstrate that bacteria, particularly *Salmonella* spp., can extensively contaminate reptile-associated environments, thereby facilitating indirect exposure during routine animal husbandry and enclosure maintenance (Cota et al., 2021; Muslin et al., 2025; Wikström et al., 2014; Zając et al., 2021). Environmental sampling may even increase the likelihood of detecting *Salmonella* compared with sampling the reptile alone, reflecting intermittent shedding and the accumulation of bacteria within the enclosure environment (Wikström et al., 2014). In the Swedish household study, *Salmonella* was detected in 50/63 (79.4%) terraria; on 49.2% cloacal swabs from reptiles and in 62.9% of environmental samples. Importantly, however, the exact same combination of subspecies and serovars was not recovered from reptile and environmental samples in any household, illustrating that serotyping alone may incompletely resolve transmission within individual terraria (Wikström et al., 2014).

Molecular evidence nevertheless demonstrates that genetically closely related *Salmonella* can occur in reptiles and their direct environment. In an ocellated lizard (*Timon lepidus*) research facility in Spain, 52% of fecal and environmental samples tested positive for *Salmonella* by real-time PCR, and PFGE analysis of cultured isolates identified indistinguishable and closely related profiles among isolates recovered from lizards and their environment, supporting horizontal transmission and environmental persistence (Martínez et al., 2011). Environmental contamination may also occur on a considerably larger scale. At reptile exhibitions in Poland, *Salmonella* was recovered from 81.2% of table and floor swabs collected after exhibitions; prevalence among sampled reptiles was 92.2% in snakes, 83.7% in lizards, and 60.0% in turtles (Zając et al., 2021).

Moreover, *Salmonella* spp. have been recovered from water bowls, glass surfaces, and soil-based substrates even weeks after routine cleaning, emphasizing the persistence of reptile-associated bacteria in the captive environment and the potential for indirect exposure during enclosure maintenance (Cota et al., 2021; Muslin et al., 2025). Observational surveys further indicate that inadequate husbandry practices, such as infrequent or inadequate cleaning and water replacement, are associated with increased *Salmonella* prevalence in reptiles and environmental contamination (Corrente et al., 2017). Consequently, indirect transmission through contaminated fomites may represent an important complement to direct reptile contact.

Evidence from human cases further demonstrates that such environmental contamination can result in infection even in the absence of reported direct reptile contact. In Australia, a 4-month-old infant developed gastroenteritis caused by *S.* Rubislaw despite having no reported direct contact with the family’s bearded dragon. The same serotype was subsequently recovered from all investigated reptile-associated environmental samples, including lizard feces, drinking water, terrarium bark and swabs, as well as the household vacuum-cleaner filter (Moffatt et al., 2010). Stronger molecular evidence for indirect transmission was obtained during an increase in *S. Tennessee* infections among infants in Germany: reptiles were kept in 8 of 16 case households, direct contact between the affected children and reptiles was denied, and identical *S. Tennessee* strains were demonstrated in child–reptile pairs from two households (Weiss et al., 2011). Household contamination may extend beyond the immediate enclosure and even contribute to foodborne exposure. In a U.S. outbreak associated with a potluck dinner, 19 cases were identified among 66 interviewed attendees. Consumption of gravy prepared by an owner of two bearded dragons was associated with illness. *Salmonella* Labadi was recovered from one of the bearded dragons as well as from household sites including a bathroom door knob and sink drains, while the outbreak PFGE subtype of *Salmonella* subsp. IV was recovered from vacuum-cleaner bag contents (Lowther et al., 2011). Together, these observations indicate that reptile-associated *Salmonella* may disseminate beyond the enclosure into the wider household environment, creating opportunities for exposure of persons who never directly handled the animal.

Feeder prey may represent an additional indirect transmission interface. In a recent surveillance study from Canada, 32 of 46 (69.6%) reptile veterinary cases with reported *Salmonella* isolation involved carnivores, and the authors identified carnivorous feeding as being associated with *Salmonella* detection. The same surveillance dataset included 13 human outbreak-associated cases linked by WGS to a 2022 *Salmonella* I 4,[5],12:i:- outbreak associated with snakes and feeder rodents (Paphitis et al., 2025). Previous outbreak investigations have likewise linked human salmonellosis to contaminated feeder rodents used for reptiles and demonstrated outbreak-related *Salmonella* in feeder rodents, reptile hosts and/or exposed humans, indicating that feeder prey may serve as both a source of infection for reptiles and a direct or indirect exposure source for reptile owners (Cartwright et al., 2016; Kanagarajah et al., 2018; Vrbova et al., 2018).

These findings complement observational evidence that husbandry practices influence environmental contamination. Inadequate or infrequent cleaning and water replacement have been associated with increased *Salmonella* prevalence in reptiles and their surroundings (Corrente et al., 2017). Consequently, indirect transmission through contaminated enclosures, household surfaces, husbandry equipment, or feeder prey may represent an important complement to direct reptile contact.

To date, however, environmental investigations have focused predominantly on *Salmonella*, whereas comparatively little information is available for other bacterial taxa. A limited number of studies have simultaneously investigated reptiles and their immediate environment for organisms such as *Pseudomonas*, *Enterococcus*, and other AMR bacteria (Mlangeni et al., 2025; Sala et al., 2019; Wimalasena et al., 2017). Furthermore, MDR *Enterococcus* spp. have been detected in commercial feed (48% MDR rate) intended for birds, fish, and reptiles, suggesting that contaminated feed may represent an additional route for the introduction and dissemination of AMR within captive reptile husbandry (Soares et al., 2023).

Public health recommendations from the U.S. Centers for Disease Control and Prevention (CDC) likewise recognize terrarium water, enclosure furnishings, feeding equipment, and surrounding surfaces as potential fomites capable of transmitting reptile-associated bacteria even in the absence of direct animal contact (CDC, 2026). Indirect exposure is not restricted to private households, as wild-caught or traded reptiles may introduce AMR bacteria into households, pet shops, breeding facilities, or aquaculture systems. Environmental persistence of resistant bacteria may additionally facilitate HGT within environmental microbial communities, further increasing the complexity of AMR dissemination (Assefa et al., 2025; Corrente et al., 2017; Unger et al., 2024).

Overall, current evidence indicates that environmental contamination represents a plausible, yet still under-investigated, pathway for human exposure to AMR bacteria associated with reptiles. Routine cleaning and disinfection of enclosures, appropriate hygiene during animal handling, and careful management of food and water sources are therefore important measures to reduce transmission risk.

At the same time, environmental sampling should become an integral component of One Health surveillance programs, as it can provide information on bacterial persistence, environmental dissemination, and potential transmission pathways and may improve detection of intermittently shed pathogens (Martínez et al., 2011; Wikström et al., 2014; Zając et al., 2025). However, outside *Salmonella*, longitudinal studies integrating paired reptile, environmental, and human isolates with whole-genome sequencing and characterization of mobile genetic elements remain scarce. Consequently, the frequency and directionality of indirect transmission pathways cannot yet be reliably quantified.

## 5 Reptiles as a food source: reptile meat consumption, AMR risk and foodborne infection

Beyond direct contact and environmental exposure, consumption of reptile-derived food products represents an additional, albeit geographically restricted, pathway for human exposure to antimicrobial-resistant bacteria. Farmed and wild-caught reptiles may harbor MDR bacteria, including ESBL-producing Enterobacterales and resistant *Salmonella* spp., thereby creating opportunities for foodborne transmission (Magnino et al., 2009; Marin et al., 2020; Mlangeni et al., 2024; Morrison, 2019; Morrison and Rubin, 2020; Ríos et al., 2022; Xia et al., 2020).

The importance of reptile meat varies considerably among regions. Consumption of snakes, turtles, and other reptiles is well established in parts of East and Southeast Asia, where reptile meat is sometimes also used for traditional medicinal purposes (Magnino et al., 2009; Vasaruchapong et al., 2017). In Africa and Latin America, bushmeat derived from reptiles contributes to food security in some rural and indigenous communities (Mazaratti et al., 2023; Ríos et al., 2022; van Vliet et al., 2015). Although reptile meat consumption is uncommon in Europe, imports of crocodilian and other exotic meats continue to attract regulatory attention (EFSA, 2007; Magnino et al., 2009). In North America, consumption of alligator and rattlesnake meat is documented in specialty markets, while imported reptile meat—including turtles, snakes, and lizards—provides an additional source of exposure (Magnino et al., 2009; Martin et al., 2000; Mishra et al., 2021; Morrison and Rubin, 2020).

Reptile meat is generally consumed after cooking, most commonly grilled, roasted, or stewed rather than eaten raw. However, contamination may occur during harvesting, slaughter, processing, storage, or food preparation, particularly in small-scale production systems or following the handling of wild-caught animals, where hygiene standards may be limited. In addition to bacterial pathogens, reptile meat has been associated with parasitic infections, including *Trichinella*, *Spirometra*, *Gnathostoma*, and pentastomids, as well as naturally occurring biotoxins (Chuboon et al., 2005; Magnino et al., 2009). Similar microbiological risks may also arise from reptiles used in traditional medicine or as aphrodisiacs.

Although documented foodborne outbreaks involving AMR bacteria from reptile meat remain rare, several studies demonstrate that reptile meat can harbor clinically important resistance determinants. A case of *Salmonella* endocarditis following consumption of rattlesnake meat illustrates the zoonotic potential of this exposure route (Mishra et al., 2021). Moreover, antimicrobial-resistant *E. coli* and *Salmonella* spp. have been recovered from reptile bushmeat sold in markets in Cameroon (Djim-Adjim-Ngana et al., 2025). In Nicaragua, black spiny-tailed iguana (*Ctenosaura similis*) meat contained ESBL-producing Enterobacterales carrying CTX-M-type β-lactamases (Ríos et al., 2022). Particularly noteworthy, reptile meat obtained from specialty markets in Canada—including turtles, snakes, and lizards—contained MDR bacteria carrying ESBL genes together with carbapenemase and colistin resistance genes *bla*_NDM-1_ and *mcr-1*, as well as numerous additional antimicrobial resistance determinants, highlighting the potential for international dissemination of clinically important ARGs through the reptile food chain (Morrison and Rubin, 2020).

Overall, the available evidence indicates that reptile meat represents a plausible, albeit incompletely characterized, route for human exposure to AMR bacteria. Given the increasing commercialization of reptile farming and international trade, reptile-derived food products should be considered within integrated One Health AMR surveillance programs, particularly in regions where reptiles are regularly consumed. Future studies combining microbiological surveillance, whole-genome sequencing, and epidemiological investigations are needed to better define contamination levels, transmission pathways, and the public health significance of these exposure routes (Djim-Adjim-Ngana et al., 2025; Morrison and Rubin, 2020; Xia et al., 2020).

## 6 Implications for public health, veterinary medicine, and One Health

The current state of knowledge across captive, wild, marine, and food-associated reptiles is summarized in Table 3.

**Table 3:** Summary of the current evidence on antimicrobial resistance in reptiles across different ecological settings.

Ecological setting	Main evidence	One Health relevance	Major knowledge gaps
Companion and captive reptiles	Frequent reports of MDR *Salmonella* and ESBL-/AmpC-producing Enterobacterales, occasional carbapenemase producers and mobile colistin resistance genes; detection of clinically relevant high-risk clones	Potential for bacterial exchange through close human-animal interfaces; veterinary care, husbandry practices, and international pet trade may influence AMR profiles	Limited longitudinal studies, scarce genomic epidemiology, and insufficient data on transmission between reptiles and owners
Wild terrestrial reptiles	Detection of AMR Enterobacterales, including *Salmonella*, and other bacteria carrying clinically relevant ARGs; reported prevalence generally lower than in captive animals	May reflect environmental AMR presence; potentially link natural ecosystems with anthropogenic AMR sources	Strong geographic bias, underrepresentation of many reptile taxa, and limited genomic characterization
Marine reptiles	Detection of MDR bacteria and clinically relevant ARGs in sea turtles and other marine reptiles, including ESBLs and occasional carbapenemases	Potential sentinels for monitoring coastal and marine AMR pollution and anthropogenic impact on aquatic ecosystems	Very limited number of studies and poor understanding of transmission pathways within marine ecosystems
Reptile-derived food products	Isolation of MDR *Salmonella* and ESBL-producing Enterobacterales, with occasional reports of carbapenemase- and colistin-resistance genes	Potential exposure route in regions, where reptile meat is produced or consumed	Limited surveillance and uncertain public health burden
Overall evidence synthesis	Reptiles harbor diverse AMR bacteria and clinically relevant resistance determinants across multiple ecological settings, but available evidence remains heterogeneous and predominantly descriptive	Reptiles represent ecologically relevant components of the One Health ecology of AMR, though their specific role in dissemination remains to be elucidated	Harmonized surveillance, whole-genome sequencing, source attribution, and transmission studies are needed to quantify the role of reptiles within AMR ecology

Building upon this synthesis, the following section discusses the broader implications of current evidence for public health, veterinary medicine, and wildlife conservation. Across captive, wild, marine, and farmed populations, reptiles harbor a broad diversity of AMR bacteria and resistance genes, including MDR Enterobacterales and *Salmonella* in particular, *Pseudomonas* spp., *Acinetobacter* spp., and *Aeromonas* spp. (Marin et al., 2020; Mlangeni et al., 2024; Sala et al., 2019; Unger et al., 2022, 2024; Valença et al., 2025). Although the magnitude of their contribution to the overall environmental AMR burden remains uncertain, these findings justify targeted hygiene practices, biosecurity measures, and risk communication to reduce opportunities for zoonotic transmission. Recommended preventive measures include regular handwashing after handling reptiles, routine cleaning and disinfection of enclosures, quarantine of newly acquired or imported animals, and safe handling of reptile meat.

From a public health perspective, *Salmonella* remains the best-documented reptile-associated zoonotic pathogen, with numerous reports linking reptile exposure to sporadic human infections and outbreaks (Table 1; CDC, 2026; Cota et al., 2021; Merkevičienė et al., 2022; Mughini-Gras et al., 2020; Paphitis et al., 2025; Waltenburg et al., 2022; Zając et al., 2021). Recent surveillance data from Canada further indicate that young children are disproportionately represented among salmonellosis cases reporting reptile or amphibian exposure, emphasizing the importance of targeted risk communication for households with young children and other susceptible persons (Paphitis et al., 2025).

However, increasing evidence demonstrates that reptiles also harbor a broad range of opportunistic bacterial pathogens carrying clinically relevant resistance determinants, including ESBL- and AmpC-producing Enterobacterales, carbapenemase-producing bacteria, mobile colistin resistance genes, multidrug-resistant *P. aeruginosa*, *Acinetobacter* spp., and globally disseminated high-risk clones such as *K. pneumoniae* ST307 and *E. coli* ST131 (Mlangeni et al., 2025; Sala et al., 2019; Schmidt et al., 2024; Unger et al., 2017; Unger et al., 2022, 2024). The presence of clinically relevant AMR bacteria in reptiles underscores their potential role in the broader One Health landscape, although the extent of their contribution to human AMR remains to be established and needs further investigations (Dégi et al., 2023; Menezes et al., 2024; Unger et al., 2024).

The role of reptiles extends beyond direct zoonotic transmission. Continuous shedding of bacteria into substrates, water, and surrounding environments facilitates interactions between reptile-associated microbiota and environmental bacterial communities. These interfaces may facilitate HGT mediated by mobile genetic elements and contribute to the persistence and dissemination of AMR within environmental bacterial populations (Bengtsson-Palme et al., 2023; Khan et al., 2021; Larsson et al., 2018; Mlangeni et al., 2024; Sala et al., 2019). However, the mechanisms, frequency, and ecological importance of HGT at these interfaces remain poorly understood and are likely to vary among habitats, husbandry systems, and geographic regions (Al-Khalaifah et al., 2025; Valença et al., 2025; Waltenburg et al., 2022).

These findings also have important implications for veterinary medicine. Antimicrobial-resistant infections complicate the treatment of captive reptiles and may reduce therapeutic success, emphasizing the importance of culture-based diagnostics and antimicrobial susceptibility testing before initiating antimicrobial therapy whenever feasible (Cristina et al., 2022; Marin et al., 2020; Merkevičienė et al., 2022). Responsible antimicrobial stewardship, together with improved husbandry conditions and infection prevention measures, should, therefore, be considered central components of reptile health management.

From a broader One Health perspective, reptiles should be incorporated into integrated AMR surveillance programs linking wildlife, companion animals, livestock, humans, and environmental reservoirs (Larsson et al., 2023; Valença et al., 2025; Waltenburg et al., 2022). Future surveillance should encompass captive, farmed, and free-ranging reptiles together with environmental sampling and integrate phenotypic antimicrobial susceptibility testing, whole-genome sequencing, and characterization of mobile genetic elements to improve source attribution and elucidate transmission pathways across ecological compartments (Mlangeni et al., 2025; Schmidt et al., 2024; Unger et al., 2024). Standardized methodologies across host species and environmental matrices will be essential for generating comparable datasets and identifying transmission networks.

While this review provides a comprehensive synthesis of the current state of knowledge, several methodological and interpretive limitations must be acknowledged. To ensure a broad and structured overview, we employed a systematic approach to the literature search and data extraction; however, given the inherent heterogeneity of the field, the synthesis maintains a narrative character to facilitate a holistic interpretation of the One Health context.

It must be noted that the current evidence base is subject to certain biases, including the restriction to the PubMed database and English-language publications. Furthermore, the data are characterized by significant taxonomic and geographic clustering—specifically a heavy focus on *Salmonella* and Enterobacterales, and a disproportionate representation of certain regions, particularly concerning wild populations and reptile-derived food products. The ecological nuances of transmission also vary significantly across settings, such as private households, breeding facilities, zoological collections, and wildlife rehabilitation centres, a complexity that current literature has yet to fully resolve.

Crucially, a major limitation of the existing literature is the inability to definitively establish transmission directionality. While the presence of shared resistance determinants is well-documented, current observational data cannot distinguish whether reptiles act as primary disseminators, recipients, or merely incidental carriers within the broader AMR landscape. Without formal source-attribution studies and quantitative assessments comparing reptiles with other environmental or animal reservoirs, the relative contribution of reptiles to the global burden of AMR remains unquantified.

Consequently, our conclusions regarding the ecological role of reptiles must remain cautious. Rather than defining reptiles as primary drivers of AMR dissemination, they should be viewed as ecologically relevant participants within the One Health interface. Future research must prioritize integrated, longitudinal, and multi-host genomic surveillance to move beyond descriptive observations and toward a mechanistic understanding of reptile-associated AMR dynamics.

## 7 Conservation implications

Conservation biology has traditionally focused on habitat loss, climate change, invasive species, and emerging infectious diseases, whereas antimicrobial resistance has only recently been recognized as an important environmental and One Health challenge affecting wildlife and ecosystems (Al-Khalaifah et al., 2025; Bengtsson-Palme et al., 2023; Berendonk et al., 2015; Larsson et al., 2023). The evidence summarized in this review suggests that AMR should increasingly be considered in reptile conservation strategies, particularly where captive breeding, translocation, and reintroduction initiatives involve close interactions among reptiles, humans, domestic animals, and the environment (Mlangeni et al., 2025; Valença et al., 2025).

Worldwide, 21% of reptile species are threatened with extinction (Cox et al., 2022). Consequently, zoological institutions, conservation breeding programs, and responsible private husbandry play a crucial role in combating biodiversity loss. The aim of this review is to provide a balanced, evidence-based assessment of antimicrobial resistance in reptiles rather than to stigmatize responsible reptile husbandry or ex situ conservation. However, the potential for reptile-associated AMR bacteria to interact with pristine ecosystems via translocation or reintroduction should be considered within biosecurity protocols, pending further research into transmission dynamics (Mlangeni et al., 2025; Valença et al., 2025; Waltenburg et al., 2022). Such measures include quarantine of newly introduced animals, evidence-based hygiene and biosecurity protocols, prudent antimicrobial use to minimize selection pressure within captive populations, and routine handwashing after direct and indirect contact with reptiles to reduce zoonotic transmission (Cristina et al., 2022).

Incorporating microbiological monitoring into wildlife health surveillance may, therefore, provide additional benefits, particularly for threatened species subject to active conservation management. Future studies should evaluate the feasibility of integrating antimicrobial susceptibility testing, whole-genome sequencing, and microbiome analyses into existing wildlife health monitoring frameworks, together with environmental monitoring of release habitats where appropriate, to better understand the risks of introducing resistant bacteria into wild populations (Larsson et al., 2023; Schmidt et al., 2024; Unger et al., 2024). Closer collaboration among conservation biologists, wildlife managers, veterinarians, microbiologists, and public health experts will be essential to ensure that future species conservation strategies account not only for host health but also for the ecological dimensions of AMR within a One Health framework. Education and training of animal caretakers, wildlife professionals, and local communities should complement these efforts by promoting evidence-based hygiene and biosecurity practices (Al-Khalaifah et al., 2025; Berendonk et al., 2015; Larsson et al., 2023; Merkevičienė et al., 2022).
